# Efficacy and safety of calcineurin inhibitor treatment for IgA nephropathy: a meta-analysis

**DOI:** 10.1186/s12882-017-0467-z

**Published:** 2017-02-13

**Authors:** Yu-Huan Song, Guang-Yan Cai, Yue-Fei Xiao, Yi-Ping Wang, Bao-Shi Yuan, Yuan-Yuan Xia, Si-Yang Wang, Pu Chen, Shu-Wen Liu, Xiang-Mei Chen

**Affiliations:** 1 Department of Nephrology, Chinese PLA General Hospital, Chinese PLA Institute of Nephrology, State Key Laboratory of Kidney Diseases, National Clinical Research Center for Kidney Diseases, 28 Fuxing Road, Beijing, 100853 People’s Republic of China; 2 Department of Nephrology, Aerospace Central Hospital, Beijing, China

**Keywords:** IgA nephropathy, Calcineurin inhibitor, Cyclosporine A, Tacrolimus

## Abstract

**Background:**

IgA nephropathy is the most common progressive glomerular disease to end stage renal failure worldwide. Calcineurin inhibitors (CNIs) is a selective immunosuppressant widely used in organ transplantation. The efficacy and safety of calcineurin inhibitors for the treatment of IgA nephropathy remain uncertain.

**Methods:**

We performed a systematic literature search using the PubMed, Embase, Science Citation Index, Ovid evidence-based medicine, Chinese Biomedical Literature (CBM) and Chinese science and technology periodicals (CNKI, VIP, and Wan Fang) for randomized, controlled trials of CNIs therapy of IgA nephropathy. Complete remission rate (CR) was defined as proteinuria less than 0.5 or 0.3 g/d. Partial remission rate (PR) was defined as proteinuria reduced to at least half of the baseline measurement and an absolute value of >0.5 or 0.3 g/d.

**Results:**

Seven relevant trials were conducted with 374 patients enrolled. CNIs plus medium/low-dose steroid had a higher CR (RR = 2.51 [95% CI,1.25 to 5.04], *P* = 0.02) compared to therapy with steroid alone or placebo, but were not significant on PR (RR = 0.87 [95% CI,0.32 to 2.38]; *P* = 0.78). Also, significant alterations were observed in proteinuria (weighted mean difference, −0.46 g/d,[95% CI:-0.55 to −0.24], *P* < 0.01) with no differences were found in serum creatinine (SCr) (weighted mean difference, 0.57,95% CI:-4.05 to 5.19; P = 0.78) and estimated glomerular filtration rate (eGFR) (weighted mean difference, 1.13,95% CI:-4.05 to 6.32; *P* = 0.34) level between the two groups. CNI therapy was associated with an increased risk for adverse events (RR = 2.21,95% CI:1.52 to 3.21, *P* < 0.01), such as gastrointestinal and neurological symptoms or hirsutism.

**Conclusions:**

CNIs might provide renal protection in patients with IgAN, but at an increased risk of adverse events. Reliably defining the efficacy and safety of CNIs in IgAN requires a high-quality trial with a large sample size.

## Background

IgA nephropathy is the most common primary glomerular disease worldwide. A wide variety of treatments have attempted to reduce kidney burden and the high risk of kidney failure events in this population. IgAN is an autoimmune kidney disease, indicating that immunosuppressive therapy may be helpful. Immunosuppressive therapy is supposed to reduce the deterioration in kidney function as well as a reduction in proteinuria. The core I β3-Gal-T-specific molecular chaperone (Cosmc) gene expression was decreased in IgAN patients. Immunosuppressive therapy can up-regulate the Cosmc expression in peripheral lymphocytes of IgAN patients. It might be the underlying mechanism of immunosuppressive therapy used in treating IgAN [1, 2]. It has been proven that calcineurin inhibitors (CNIs) which include cyclosporine A (CsA) and tacrolimus (TAC), can suppress the immune response by downregulating the transcription of various genes in T cells.

There are only a few small studies available using CNIs for the treatment of IgAN ten years ago [3], mainly affected by the very first report that discouraged the use of this medication in IgAN due to an increase in serum creatinine (SCr), although the complication was reversible [4]. From then on, due to the lack of controlled clinical trials, the benefit and risk of CNIs in the treatment of IgAN remained uncertain [5–8]. Recently, several randomized controlled trials (RCTs) suggested that CNIs might be effective for IgAN. Moreover, there are a few other studies that have successfully used CNIs in resistant IgAN patients, which demonstrated that CNIs could decrease proteinuria in IgAN patients who showed resistance to steroids and/or other immunosuppressants [9]. We therefore conducted this meta-analysis of all available RCTs to comprehensively ascertain the benefits and risks of CNI treatment in comparison with steroids or placebos in patients with IgAN.

## Methods

### Identification of eligible studies

Two researchers (GYC and YHS) performed a systematic literature search using the PubMed, Embase, Science Citation Index, Ovid evidence-based medicine, Chinese Biomedical Literature (CBM) and Chinese science and technology periodicals (CNKI, VIP, and Wan Fang) databases without any language restriction. All of the relevant studies were published between 1986 and July 2016. The following key words and subject terms were used in the search: ‘IgA nephropathy’, ‘immunoglobulin A nephropathy’, ‘IgA nephritis, ‘IgA glomerulonephritis’, ‘Berger’s disease’, ‘cyclosporine A’, ‘CsA’, ‘tacrolimus’, ‘FK506’, and their derivative words.

### Inclusion and exclusion criteria

Two authors independently selected information from the studies and disagreement was resolved by consensus. The titles and abstracts were scanned to exclude any trials that were clearly irrelevant in the first stage. The full texts of the relevant articles were read in order to determine whether they contained information on the topic of interest in the second stage. The baseline data of patients, proteinuria level, doses and duration of CNIs use, follow-up duration, clinical parameters and adverse events were included in the extracted information.

Inclusion criteria consisted of: (1) the study design was a RCT; (2) the study focused on patients with biopsy-proven IgA nephropathy; (3) the study compared TAC or CsA with corticosteroid or placebo in the induction therapy of IgAN; and (4) at least one of the following outcomes was reported: the complete remission (CR) or partial remission (PR) of proteinuria, changes of clinical outcomes (including proteinuria, serum creatinine or eGFR) and adverse events.

CR was defined as proteinuria less than 0.5 or 0.3 g/d and a normal serum creatinine (Scr) level. PR are among those patients who did not have a CR, was defined as proteinuria reduced to at least half of the baseline measurement and an absolute value of >0.5 or 0.3 g/d and as well as a relatively stable Scr level (variation less than 25%).

Exclusion criteria were: (1) did not including English abstract; (2) studies including minors; (3) did not describe the numbers of patients who recovered, deteriorated, or had renal replacement treatment clearly.

### Assessment of trial quality

We assessed the quality of RCTs using a standard scoring system proposed in the Jadad scale criteria [10]. These included: (1) whether the randomization method was appropriately performed; (2) whether double-blindness was used in the RCT and whether it was appropriate; (3) whether the report (the patient number and reasons) of withdrawal and drop-outs was stated clearly. We classified the RCTs as high quality if they scored >2. Otherwise, assessed them as low quality [11].

### Statistical analysis

Cochrane RevMan 5.3 was used to perform statistical analyses. The results were stated as relative risks (RR), for dichotomous outcomes, and weighted mean differences, for continuous outcomes, with 95% confidence intervals (95% CI). The heterogeneity Q statistic test was used to analyze heterogeneity among the included trials. If it indicated heterogeneity (*p* < 0.05) across trials, the DerSimonian and Laird method in the random effect model was selected. Otherwise, the Mantel-Haenszel method in the fixed-effect model was used.

## Results

### Studies included in the meta-analysis

**Fig. 1:**
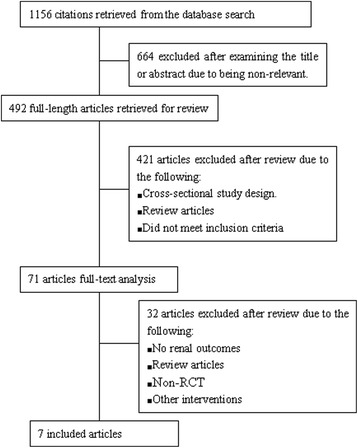
Study selection process

The comprehensive literature retrieval yielded 1156 articles. Of these, 71 were acquired in full-text form. Seven RCTs were identified as appropriate for inclusion in this meta-analysis (Fig. 1). The included studies provided information on a total of 374 patients. Table 1 showed the summarized characteristics of the included studies. Among them, Six studies (334 patients) included IgAN patients with proteinuria 1–3.5 g/d [4, 12–16]. One study (40 patients) included patients mainly with mild or moderate proteinuria [17]. The Jadad method was assessed for the quality of the RCTs. It ranged from 2 to 7 and two trials were of high quality (Jadad score = 7).

**Table 1:** Main Characteristics of the included studies

Studies	Patients	Sample size	CNIs group	Follow-up (mo)	Control	Definitions of PR and CR	Drop-in (CNI/control)	ACEI/ARB	Jadad score
Lai 1987 [4]	proteinuria ≥ 1.5 g/day, eGFR > 50 ml/min/1 73 m^2^	19 (9/10)	CsA 5 mg/kg/day, then reduced by increments of 25% every 4 days over 2 weeks	3/6	placebo	CR: Proteinuria less than 0.5 g/day. PR: proteinuria reduced to at least half of the baseline measurement and an absolute value of >0.5 g/day).	1/0	--	7
Liu 2014 [15]	proteinuria >1.0 g/day eGFR > 30 mL/min/1.73 m^2^	48 (24/24)	CsA 3 mg/kg/day with low-dose MP (8 mg/day). Twelve weeks later, the dose was gradually reduced by 50 mg every month then maintained at a maintenance dose of 25 mg/day.	12/12	MP 0.8 mg/kg/day (max 48 mg/day)	CR: Proteinuria less than 0.3 g/day. PR: proteinuria reduced to at least half of the initial level and an absolute value of >0.3 g/day).	3/1	losartan (50 mg/day)	3
Xu 2014 [16]	proteinuria >1.0 g/day <3.5 g/day eGFR ≥ 60 mL/min/1.73 m^2^ or scr < 150umol/L	96 (48/48)	CsA 3 mg/kg/day for 3 months, then 2 mg/kg/day for 9 months; PDN 0.6–0.8 mg/kg/day (max 40 mg/day), then tapered	12/12	PDN 1 mg/kg/day (max 60 mg/day)	CR: proteinuria < 0.5 g/day, serum albumin > 35 g/L, and normal Scr. PR: proteinuria reduced by >25% but still higher than 0.5 g/day, and stable Scr.	0/0	Valsartan 80–160 mg/day	3
Kim 2013 [17]	eGFR >45 mL/min/1.73 m^2^ or scr < 1.5 mg/dl UACR ≥ 0.3 and <3 g/g creatinine	40 (20/20)	TAC 0.1 mg/kg/day, After 8 weeks reduced to 0.05 mg/kg/day	3/3	placebo	--	1/0	ARB10/20,8/20	7
Zhang 2013 [14]	proteinuria >1.0 g/day <3.5 g/day eGFR >60 mL/min/1.73 m^2^ or scr < 221umol/L	25 (11/14)	TAC 0.075 mg/(kg · d), then tapered. PDN 30 mg/day, then tapered	6/6	PDN 0.5 mg/kg/day (max 60 mg/day)	CR: proteinuria < 0.3 g/day, and normal Scr. PR: proteinuria reduced by >30% but still higher than 0.3 g/24 h, and stable Scr.	0/0	--	2
Ou-yang 2015 [13]	eGFR >45 mL/min/1.73 m2 or scr < 1.5 mg/dl UACR ≥ 0.3 and <3 g/g creatinine	56 (28/28)	CsA 100 mg/day, then tapered, MP 0.5 mg/kg/day (max 36 mg/day)	6/6	MP 0.8 mg/kg/day (max 48 mg/day)	CR was defined as proteinuria < 0.3 g/24 h, and normal Scr. PR was defined as proteinuria reduced by >30% but still higher than 0.3 g/24 h, and stable Scr.	1/0	losartan (100 mg/day)	7
Liu 2015 [12]	proteinuria >3.5 g/day scr < 150umol/L	90 (45/45)	CsA 100 mg/day; PDN 1 mg/kg/day, then tapered	3/3	PDN 1 mg/kg/day, then tapered	--	0/0	--	2

Drop in, patients who are randomized to standard/control arm but start taking/using the experimental treatment; *Scr* serum creatinine, *MP* methyl prednisolone, *PDN* prednisolone, *UACR* urine albumin to creatinine ratio, *CsA* Cyclosporine A, *TAC* Tacrolimus, *CNI* Calcineurin inhibitor, *PR* Partial remission rate, *CR* Complete remission rate

### Trial outcomes

#### Effect on Proteinuria

**Fig. 2:**
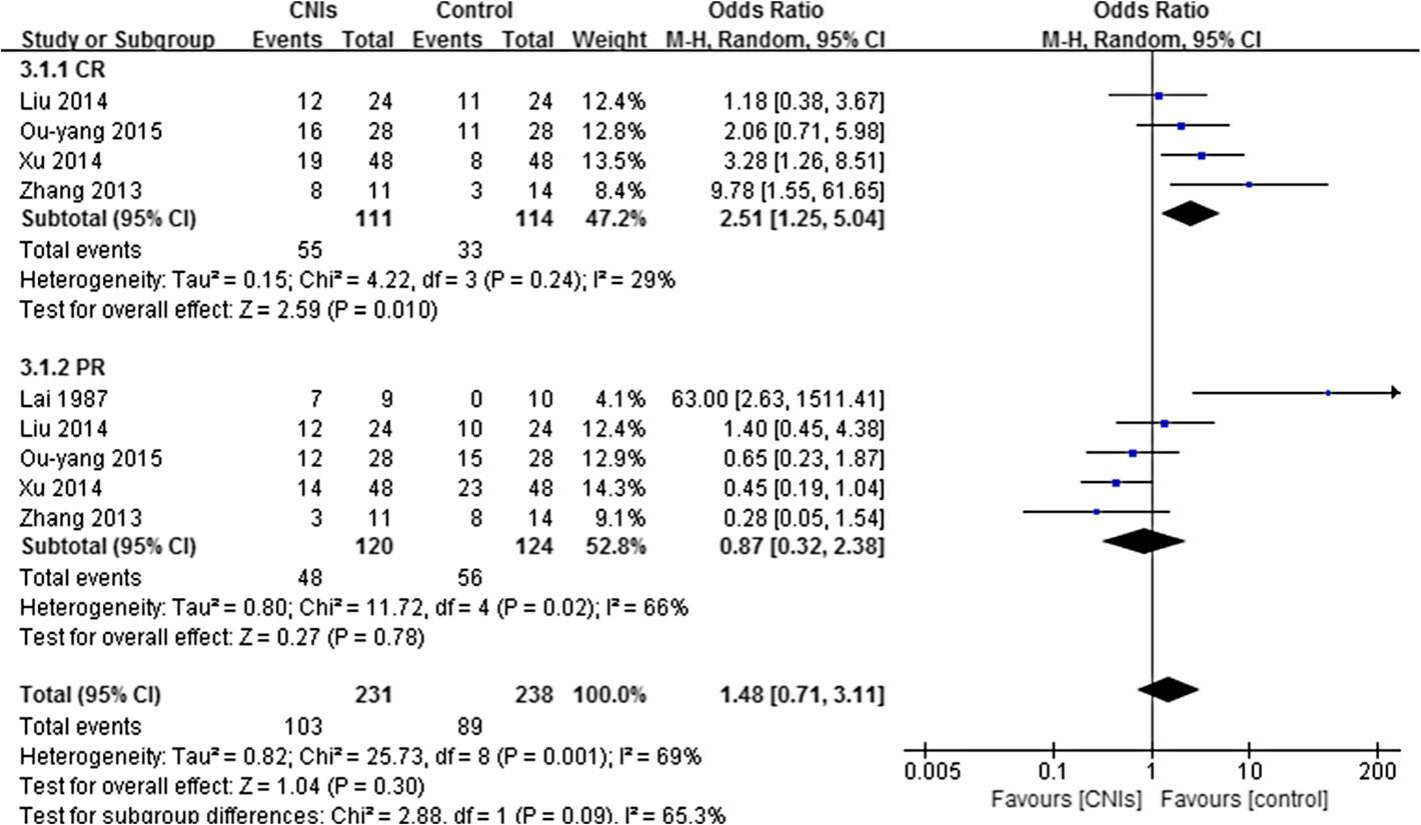
Forest plot of the relative risks for CR and PR for CNIs versus steroid alone or Placebo in the treatment of IgAN

Five studies assessed CR or PR in a total of 225 patients, CR (RR = 2.51; 95% CI 1.25 to 5.04) occurred more frequently among people in the CNIs group compared with the control group. PR did not reach a significant difference between CNIs and steroid alone or Placebo (RR = 0.87, 95% CI 0.32 to 2.38) (Fig. 2).

**Fig. 3:**
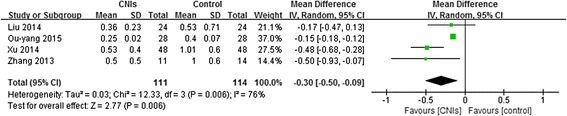
Forest plot of the Effect of CNIs for proteinuria (g/d) at the end of treatment or during follow-up

Three studies assessed urinary protein excretion in a total of 169 patients. 24-h proteinuria was significantly lower in patients using CNIs than in the control groups (weighted mean difference, −0.46 g/d, 95% CI:-0.55 to −0.24) at the end of treatment or during follow-up (Fig. 3). The randomized effects model was selected because heterogeneity was significant (*P* < 0.01).

#### Effect on Scr and eGFR

**Fig. 4:**
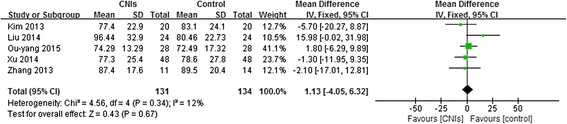
Forest plot of the Effect of CNIs on eGFR at the end of treatment or during follow-up

**Fig. 5:**
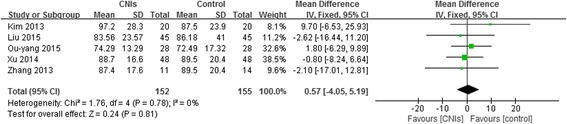
Forest plot of the Effect of CNIs on SCr at the end of treatment or during follow-up

There were five studies assessed eGFR or Scr in patients. Both eGFR (weighted mean difference, 1.13,95% CI:-4.05 to 6.32 *P* = 0.34) (Fig. 4) and Scr (weighted mean difference, 0.57,95% CI:-4.05 to 5.19, *P* = 0.78) (Fig. 5) were not significantly lower in patients using CNIs than in the control groups at the end of treatment or during follow-up. The fixed effects model was used because heterogeneity was not significant (*P* = 0.78).

#### Adverse events

**Fig. 6:**
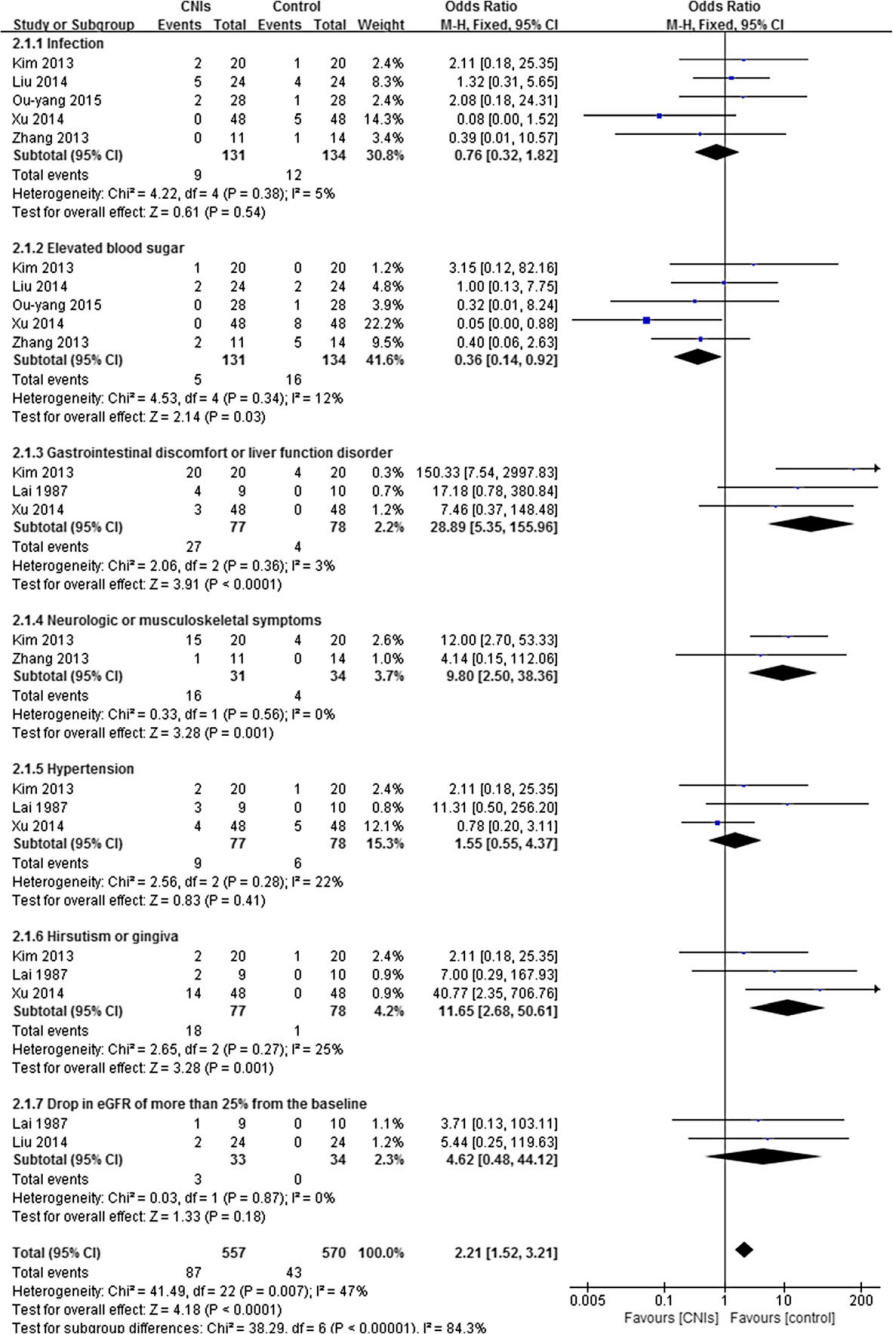
Forest plot of the relative risks for adverse events at the end of treatment or during follow-up

All adverse events were collected which mentioned in the included articles, and the most prevalent events were analyzed in the synthesis. The following outcomes were included: liver function disorder, respiratory symptoms, such as infection, elevated blood sugar, cardiovascular symptoms, such as hypertension, eyesight degradation, hirsutism, gingivitis, musculoskeletal symptoms, gastrointestinal discomfort, neurologic discomforts, hematologic symptoms, drop in eGFR, urinary tract infection, and withdrawal. Data on adverse events potentially caused by treatment were collected from the RCTs (Fig. 6).

CNIs therapy was associated with an increased risk for several events (RR, 2.21 [95% CI, 1.52 to 3.21]). Significantly, patients receiving CNIs appeared to have a higher risk of experiencing gastrointestinal discomfort or liver function disorder (RR, 28.89 [95% CI, 5.35 to 155.96]), neurologic or musculoskeletal symptoms (RR, 9.80 [95% CI, 2.50 to 38.36]), and hirsutism or gingivitis (RR, 11.65 [95% CI, 2.68 to 50.61]). However, fewer patients who received CNIs developed elevated blood sugar (RR, 0.36 [95% CI, 0.14 to 0.91]). Hypertension and drop in eGFR of more than 25% from the baseline did not reach a significant difference between CNIs and steroid alone or placebo. The fixed-effects model was selected because heterogeneity was undetectable when the effect sizes of side effects were evaluated (*P* > 0.05).

In 7 RCTs, there were five patients in CNI groups and one patient in control group who withdraw from the therapy. One patient showed irreversible kidney failure even with a dosage reduction of CsA and treatment was subsequently stopped [4]. Three patients in the CsA group and one patient in the control group developed severe pneumonia in the second to third month of treatment, after which the steroid and CsA therapies were discontinued [15]. One patient was advised to discontinue the TAC after the fourth week of treatment because of general weakness and myalgia [17].

#### Publication bias

**Fig. 7:**
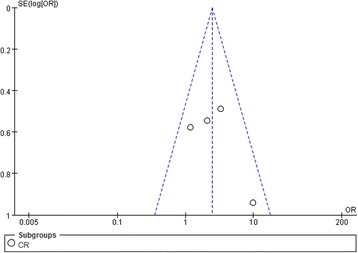
Funnel plot of four RCTs for Effect in proteinuria CR of CNIs treatment of IgAN patients

**Fig. 8:**
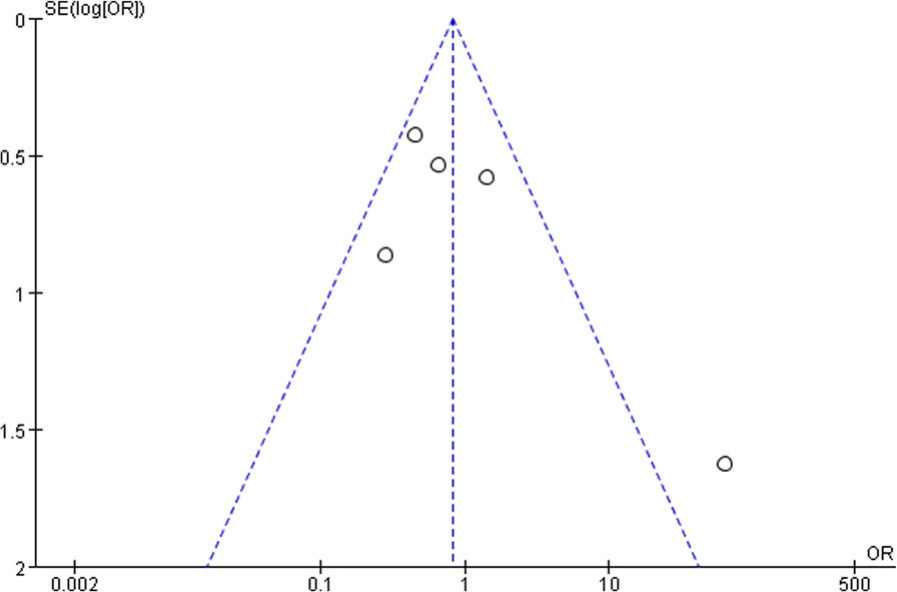
Funnel plot of five RCTs for Effect in SCr of CNIs treatment of IgAN patients

The funnel plots exhibited symmetric patterns for both proteinuria and renal function, as shown in Figs. 7 and 8. Because the sample sizes of the 7 RCTs included in this meta-analysis were all small, we conducted Begg’s test to evaluate the publication bias using Stata software, which indicated no significant heterogeneity in the 7 RCTs.

## Discussion

IgAN is the most common type of glomerulonephritis worldwide [18, 19]. It is now known to slowly progress to end-stage renal disease (ESRD) [20–23]. Proteinuria is one of the strongest independent prognostic factors [24, 25]. IgAN with severe proteinuria are conventionally subjected to treatment with various immunosuppressive regimens with conflicting results [26, 27]. Studies showed that immunosuppressive therapy for IgAN may reduce the risk of ESRD by 70% compared with supportive therapy after > 5-year follow-up [28, 29]. CNIs are widely used as immunosuppressive drugs. Studies suggest that CNIs are effective in decreasing proteinuria in a variety of glomerular diseases, including IgAN [30, 31].

So far, few RCTs have analyzed the role of CNIs in patients with IgAN. The current meta-analysis of seven trials involving 374 patients with IgAN revealed that the combination of CNIs and medium/low-dose steroid was more effective in reducing proteinuria compared with the steroid group alone, suggesting a synergistic effect between CNIs and steroid. Similar to our findings, several studies also indicated that patients with IgAN could experience significant improvement in proteinuria and hypoalbuminemia during CNI treatment [32]. In addition, the risk of developing elevated blood sugar appeared lower in patients treated with CNIs in comparison with placebo or steroid. Moreover, this meta-analysis concluded that there was no significant difference in the risk of renal impairment or rate of decline of eGFR between two groups.

CNIs were associated with a higher incidence of experiencing gastrointestinal discomfort or liver function disorder, and neurological or musculoskeletal symptoms than placebo or steroid. They were also associated with a higher incidence of experiencing hirsutism or gingivitis. This was consistent with the results of studies containing CNIs [33].

IgA patients who achieved remission had far better outcomes than those who never achieved remission [34, 35]. These findings suggest that achieving remission, whether CR or PR, is important in IgA patients to improve renal survival, irrespective of glomerular disease type. In current systematic review, CNIs group increased the rates of CR compared with steroid alone or placebo.

There are a few other studies that have successfully used CsA or TAC in resistant IgAN patients. In one retrospective case series of 13 adult patients with IgAN and significant proteinuria, more than half of the patients did respond to CsA therapy with or without steroids, with long-term remission. A rise in Scr was observed in only two patients, and was mild and reversible in these cases [36]. In a non-randomized study, Chabova and colleagues administered 5 mg/kg/day of CsA plus alternate day 5–10 mg prednisolone to 6 IgAN patients with nephrotic-range proteinuria and normal Scr, who were resistant to three months of glucocorticoid therapy [37]. They aimed for a trough serum cyclosporine level of 70–150 ng/mL and continued the regimen for one year. After one month of treatment, proteinuria reduced from 4.66 ± 0.43 g/day to 1.38 ± 0.29 g/day, and after one year to 0.59 ± 0.14 g/day. GFR did not differ significantly from the baseline in two years. In a retrospective study by Shin and colleagues on 14 children with IgAN, a significant decrease in proteinuria and increase in serum albumin concentration without any rise in Scr level was observed [38]. A decrease in histologic grade of IgAN was seen in a follow-up biopsy of 50% of the patients. These researchers suggested that CsA has a significant role in decreasing proteinuria and reversing kidney pathology in children with IgAN. In another interesting recent study, remission of nephrotic-range proteinuria could be induced in 9 of 11 IgAN patients with the use of TAC, which was explained through the effect of the drug in podocyte cytoskeleton stabilization through inhibition of calcineurin expression [39].

However, there is still a strong debate regarding the use of CNIs, especially CsA for the treatment of proteinuria in IgAN, mainly due to concerns about the possible increase in Scr caused by CsA, although it is being used as one of the main immunosuppressive agents in various other proteinuric glomerulonephritides. It concluded that there was no significant difference in the risk of ESRD or rate of decline of GFR in patients treated with CsA or placebo in a meta-analysis.

Our meta-analysis had four limitations. First, the proteinuria outcomes were measured while on CNI, whether a reduction in proteinuria while on a CNI will be sustained or will rebound after the CNI is stopped is not certain. We should also address the limitation of using proteinuria as a surrogate outcome measure, and the implication of rebounding proteinuria after stopping CNI. Second, the renal outcomes that were assessed were over likely too short a time period to see any beneficial or detrimental effects from chronic CNI use. Our meta-analysis do not show significant benefit on kidney function, as serum creatinine or eGFR. Long-term, large sample, multicenter RCTs are needed to confirm the efficacy and safety of CNIs in the treatment of IgAN. Third, the number of subjects included in this analysis was not particularly great. Finally, there appears to be lack of published small studies with negative outcomes. The risk of publication bias in which studies with negative results is also a limitation.

The current meta-analysis was generally consistent with these reviews [7, 8]. Thus, we believe that the results of our studies can help to prevent the discouragement of the use of this medication for an idiopathic immunologic disease without many therapeutic choices. The fear of increase in Scr seems to have prevented the researchers from designing clinical trials to study this valuable immunosuppressive agent in the treatment of IgAN, and we suggest starting such trials for a better long-term judgment.

## Conclusions

Prescription of CNI combined with medium-dose steroid resulted in significant reduction in proteinuria without deteriorated renal function, showing that CNI may be a promising agent for IgAN. It is important to use the lowest effective dosage of CNIs and monitor its level closely because of possible complications. Larger RCTs of CNIs which are sufficiently powered to evaluate patient-relevant end points, including adverse events, and that examine the optimal duration of treatment are now required in IgAN patients with a range of kidney function.

## Abbreviations

95% CI: 95% confidence intervals
CNIs: Calcineurin inhibitors
Cosmc: Core I β3-Gal-T-specific molecular chaperone
CR: Complete remission
CsA: Cyclosporine A
ESRD: End-stage renal disease
IgAN: Immunoglobulin A nephropathy
PR: Partial remission
RCT: Randomized controlled trial
RR: Relative risks
Scr: Serum creatinine
TAC: Tacrolimus
